# Extended Intake of Mulberry Leaf Extract Delayed Metformin Elimination via Inhibiting the Organic Cation Transporter 2

**DOI:** 10.3390/pharmaceutics12010049

**Published:** 2020-01-07

**Authors:** Hyun Wook Huh, Young-Guk Na, Ki-Hyun Bang, Sung-Jin Kim, Minki Kim, Kyung-Tae Kim, Jong-Seong Kang, Young-Ho Kim, Jong-Suep Baek, Hong-Ki Lee, Cheong-Weon Cho

**Affiliations:** 1 College of Pharmacy, Chungnam National University, Daejeon 34134, Korea; hhw3573@nate.com (H.W.H.); youngguk@cnu.ac.kr (Y.-G.N.); robotkr@nate.com (K.-H.B.); lanop@naver.com (S.-J.K.); zkzkang@naver.com (M.K.); kangjss@cnu.ac.kr (J.-S.K.); yhk@cnu.ac.kr (Y.-H.K.); 2 Food Science and Technology Major, Division of Applied Bioengineering, College of Engineering, Dong-Eui University, Busan 47340, Korea; kimkkt@deu.ac.kr; 3 Department of Herbal Medicine Resource, Kangwon National University, Samcheok 25949, Korea; jsbaek@kangwon.ac.kr

**Keywords:** diabetes mellitus, metformin, *Morus alba* L., mulberry, pharmacokinetics, pharmacodynamics, organic cation transporter 2

## Abstract

Diabetes mellitus (DM) has become a major health problem in most countries of the world. DM causes many complications, including hyperglycemia, diabetic ketoacidosis, and death. In Asia, mulberry has been used widely in the treatment of DM. Combination of drugs with herbal medicine may reduce the unwanted side effects caused by drugs. In this study, the influence of extended mulberry leaves extract (MLE) intake on metformin (Met) was evaluated in terms of pharmacokinetics and pharmacodynamics in DM-induced rats. Three week-treatment of MLE alone produced the anti-hyperglycemic effect (around 24%) if compared to the control. Interestingly, Met administration after MLE treatment for 3 weeks enhanced about 49% of the anti-hyperglycemic effect of Met. In addition, the extended intake of MLE potentiated the anti-hyperglycemic effect of Met on various concentrations. This potentiated anti-hyperglycemic effect of Met appears to be due to the pharmacokinetic change of Met. In this study, 3 week-treatment of MLE reduced the elimination of Met in DM-induced rats. In addition, MLE reduced the human organic cation transporter 2 (hOCT2) activity in a concentration-dependent manner. Thus, these findings suggest that MLE lowered the elimination of Met via inhibiting the hOCT2.

## 1. Introduction

Diabetes mellitus (DM) is a major health problem in most countries of the world. According to the World Health Organization (WHO) [1], 422 million peoples are diagnosed as DM, and DM caused 1.5 million death in 2012. DM is characterized by metabolic abnormalities along with symptoms, including hyperglycemia, diabetic ketoacidosis, polyuria, polydipsia, polyphagia, and loss of pancreatic β-cells [2]. In particular, hyperglycemia can cause severe damage to the blood vessels, which leads to cardiovascular disorders, hepatopathy, nephropathy, and neuropathy [3].

Five classes of anti-hyperglycemic drugs—including sulfonylurea, biguanides, α-glucosidase inhibitor, meglitinides, and thiazolidinediones—are available for the treatment of DM [4]. Metformin (Met) is a biguanide anti-hyperglycemic drug and is the front-line drug in the management of diabetes in patients. Met reduces blood glucose levels by decreasing glucose output from the liver and by increasing peripheral insulin receptor sensitivity without affecting insulin secretion [5]. However, an endocrine disorder, such as DM, that requires the extended treatment can produce unwanted side effects, including weight gain, liver, and kidney dysfunctions during the extended treatment. Thus, a new approach is needed to decrease the side effects of drugs. The combination therapy is a common approach to enhance the efficacy of the drug and to reduce the unwanted side effects for DM control.

Plants have been used as a therapeutic purpose in many Asian countries, and many drugs are derived from plants. Mulberry, *Morus alba* L., is the common deciduous tree in Asia. Its products, such as fruits, leaves, and roots have been used traditionally as an anti-hyperglycemic folk remedy in the Asian country. Anti-hyperglycemic effects of mulberry have been frequently reported in literatures [1,6,7,8,9,10]. Although the mechanisms of anti-hyperglycemic actions of mulberry have been less well understood, it has been known that the anti-hyperglycemic effects are related with ingredients, such as caffeic acid, syringaldehyde, chlorogenic acid, and rutin of mulberry [11,12,13,14,15,16,17].

Combinations of drugs with herbal medicine may reduce the unwanted side effects caused by drugs. For instance, cinnamic acid derivatives when combined with Met reduced the expression of fatty acid synthase and β-Hydroxy β-methylglutaryl-CoA (HMG CoA) reductase involved in the secondary complications of DM [18,19]. However, these combinations may produce three different types of effects—namely synergistic, additive, and antagonistic [5]. In addition, it is reported that drug interactions with herbal medicines showed various behaviors [20,21,22,23]. In particular, the pharmacokinetic herb-drug interactions related to drug absorption, distribution, metabolism, and elimination, lead to increased or decreased plasma levels of drugs and affect drug efficacy [20,21,22,23]. For examples, *Hypoxis hemerocallidea* and l-canavanine inhibited the P-glycoprotein (P-gp) efflux of nevirapine in the Caco-2 cell [24]. Capsaicin inhibited the P-gp activity in KB-C2 cell and had the potential to induce P-gp in human [25,26]. While the St John’s wort lowered the disposition of sulfonylureas by induction of various hepatic CYP enzymes, the Cassia inhibited the activities of various hepatic CYP enzyme which metabolize the anti-hyperglycemic drugs, such as glibenclamide, glimepiride, glipizide, and nateglinide [27,28]. Moreover, herbal medicines can alter renal drug elimination. It was reported that *Houttuynia cordata* ethanol extract reduced renal excretion of Met via decrease of organic cation transporters (OCT) uptake of Met [23]. Thus, before applying a combination with herbal medicine, it is critical to evaluate the pharmacokinetic and pharmacodynamic profiles of the drug co-administered with herbal medicine. This study aims to evaluate the influence of extended *Morus alba* L. extract intake on Met in terms of pharmacokinetics and pharmacodynamics in DM-induced rats.

## 2. Materials and Methods

### 2.1. Chemicals and Plant Materials

Streptozotocin (STZ, purity ≥ 98%) and phenformin (internal standard, IS, purity ≥ 98%) were purchased from Sigma-Aldrich (St. Louis, MO, USA). Met (purity ≥ 98%) was a gift from the Korea United Pharm Inc. (Seoul, Korea). High-performance liquid chromatography (HPLC) grade acetonitrile (ACN) and methanol (MeOH) were purchased from JT baker (Phillipsburg, NJ, USA). All the other agents were of analytical grade and were obtained by commercial sources. The mulberry leaf extracts (MLE) were obtained from the MSC (Yangsan, Korea). Briefly, *Morus alba* L. was added into purified water and then extracted under the pressure at 121 °C for 3 h. These extracts were fermented by adding 1% (*w*/*v*) enzyme (Viscozyme^®^) under pH 5.0 at 45 °C for 15 h. MLE was dried using a freeze-dryer [29]. MLE used in this study included phytochemicals such as trans-caffeic acid, syringaldehyde and chlorogenic acid that have been known to increase glucose uptake into skeletal muscle cells. In MLE, trans-caffeic acid was the substance with the highest content (about 0.7 mg/g).

### 2.2. Animals

Male Sprague–Dawley rats were used in this study. Rats aged 7 weeks were obtained by Samtako (Osan, Korea), and they were housed at 22 °C with a 12-h light–dark cycle and given free access to food and water. Before the experiments, animals were acclimated for 1 week. All experiments were performed according to the guidelines established by the Chungnam National University Institutional Animal Care and Use Committee. This study was approved by the Local Ethical Committee of Chungnam National University (protocol no. CNU-01150, 19 October 2018).

### 2.3. Induction of DM

DM was induced by an injection of STZ. Animals were fasted overnight and then STZ was injected via tail vein at a dose of 45 mg/kg. To overcome the acute hypoglycemia, 5% glucose solution was provided a day. Animals were fasted at least 8 h before collecting whole blood via jugular vein, and then fasting blood glucose level was measured using a blood glucose meter (Accu-Chek^®^ Active, Hoffmann-La Roche, Basel, Switzerland). Glucose level was measured at 0, 1, and 5 days post-injection of STZ. Fasting glucose level above 200 mg/dL was set as the criteria of DM.

### 2.4. Anti-Hyperglycemic Effect of Repeated Administrations of MLE

To evaluate the anti-hyperglycemic effect of repeated administrations of MLE, MLE was orally administered at a single dose of 600 mg/kg for 21 days. As a negative control, saline was administered for 21 days. The dose of MLE was selected by the preliminary study. According to the literature [30], the dosage of MLE at a single dose of 600 mg/kg/day for 49 days was produced the antidiabetic effect and well-tolerated in animals. Blood samples were collected on 0, 2, 4, 6, 9, 12, 15, 18, 21, 22 days and the fasting glucose levels were measured. Fasting glucose level was converted to the relative blood glucose level by a following Equation (1)

(1) $$\mathrm{Relative}\;\mathrm{blood}\;\mathrm{glucose}\;\mathrm{level}\;=\;\frac{\mathrm{Fasting}\;\mathrm{blood}\;\mathrm{glucose}\;\mathrm{level}\;\mathrm{at}\;\mathrm{time}\;\mathrm{points}}{\mathrm{Fasting}\;\mathrm{blood}\;\mathrm{glucose}\;\mathrm{level}\;\mathrm{at}\;0\;h}\times \;100\;(\%)$$

Anti-hyperglycemic effect was estimated using an area under curves of the relative blood glucose level versus time curves (AUG). AUG was calculated by a trapezoidal rule.

### 2.5. Impact of Repeated Administrations of MLE on the Anti-Hyperglycemic Effect of Various Doses of Met

To evaluate the influence of repeated administrations of MLE on the anti-hyperglycemic effect of various doses of Met, the MLE was orally administered for 21 days and then the increasing doses of Met were orally administered on day 22. Briefly, animals orally received saline or MLE (600 mg/kg) of the same volume for 21 days. On day 22, animals received the increasing doses of Met (25, 50, and 100 mg/kg). Fasting glucose level was measured at 0, 2, 4, and 12 h post-administration of Met. AUG was calculated as described in Section 2.4. The AUG value corresponding to each dose of MET was analyzed by linear regression and the slope was evaluated. Statistical differences of slope between MLE and saline treatment group was assessed by the Student’s *t*-test.

### 2.6. Influence of Repeated Administrations of MLE to the Pharmacokinetics of Met

Pharmacokinetic interaction between herb and drug has been reported in many studies. To evaluate the interaction between the MLE and Met, pharmacokinetic analysis of Met was assessed after the repeated administrations of MLE or saline. Briefly, MLE (600 mg/kg, PO, orally once a day) or saline was orally administered to animals for 21 days, then the animal received the Met per oral at a dose of 50 mg/kg. After the administration of Met, the blood samples were taken via jugular vein at 0.5, 1, 1.5, 2, 4, 6, 8, 12, and 24 h. Blood samples were centrifuged at 1000× *g* for 10 min and the plasma was stored at −70 °C. The plasma samples were analyzed by HPLC with a UV detector. PK parameters for oral administration were determined as a non-compartmental model using WinNonlin 5.3.1 software (Pharsight, Princeton, NJ, USA).

### 2.7. Interference of MLE to Met Analysis in Rat Plasma

To evaluate the interfering effect of MLE presence to Met analysis in rat plasma, the Met-spiked samples with the MLE (10 µg/mL) in rat plasma were used and the validation parameters such as accuracy and precision were evaluated in the presence or absence of MLE in rat plasma.

For the calibration curve, the peak area ratios of Met (0.2, 0.4, 0.8, 1, 2.5, 5, and 10 µg/mL) to IS were plotted against corresponding nominal concentrations of Met. The linearity of the regression curve in the range 0.4–10 µg/mL was assessed on the base of the residual plot. Concentrations of the samples were interpolated from the regression curve. The correlation coefficient of the calibration curve was >0.99.

Inter- and intra-day reproducibility was measured to assess accuracy and precision using quality control (QC) samples (low QC, LQC, 0.5 µg/mL; medium QC, MQC, 1 µg/mL; high QC, HQC, 5 µg/mL). The relative error (RE) and relative standard deviation (RSD) were used to evaluate the accuracy and precision of the bioanalytical method. The ±15% fixed range applied as an acceptance of criteria.

In addition, to assess the interference in the chromatogram from the MLE at the retention time of Met, the chromatogram of plasma samples obtained from rats received the MLE for 21 days was analyzed.

### 2.8. Effects of MLE on Met Uptake in HEK-293 Cells Overexpressing hOCT2

Human embryonic kidney cells (HEK-293 cells) over-expressing hOCT2 were used to access the changes in Met uptake by MLE. HEK-293 cells over-expressing hOCT2 was obtained from ThermoFisher Scientific (Waltham, MA, USA). Dulbecco’s modified Eagle’s medium (DMEM) with 10% FBS, 100 µg/mL of streptomycin, and 100 U/mL of penicillin was used for culture of the cell. The cell was humidified at 37 °C with 5% CO_2_ atmosphere.

Briefly, HEK-293 cells over-expressing hOCT2 were located into the poly-d-lysine coated 24-well plates (5.0 × 10^5^ cells/well). DMEM with 10% fetal bovine serum was added into the plates and incubated for 24 h. After the incubation, cells were washed twice using prewarmed Hank’s balanced salt solution (HBSS). Prewarmed HBSS containing Met (2.5 µM) with or without MLE (ranged from 0.1 to 100 µg/mL) was added into the plates. For the positive control, metoprolol (MP, 25 µM) was used as the hOCT2 inhibitor. Plates were incubated for 10 min and cells were washed with iced blank HBSS. Then, the 150 μL of 1 N NaOH was added into the cell to lysis the cell. After 15 min of standing, 150 μL of 1 N HCl was added into the plates and cells was harvested. The lysates were centrifuged at 122,000× *g* rpm for 10 min. The concentration of Met was measured by LC-MS/MS.

### 2.9. Hepatotoxicity of the Extended Intake of MLE

To evaluate the hepatotoxicity of the extended intake of MLE, the serum biochemical parameters including alkaline phosphatase (ALP), alanine aminotransferase (ALT) were measured after the administration of MLE (600 mg/kg, oral) for 21 days. In brief, the rat was received the MLE (600 mg/mL, oral) or saline for 21 days. On day 0 and 21 days, the whole blood was collected via jugular vein and then stored at room temperature for 10 min. Then, blood samples were centrifuged at 1000× *g* for 10 min and serum (supernatant) was separated. Serum biochemical parameters (ALP, alkaline phosphatase; ALT, alanine transaminase) were measured using a VetScan VS2 (Abaxis, Union city, CA, USA).

### 2.10. HPLC and LC-MS/MS Analysis

To extract the Met in plasma samples, the previously described method was partially validated using a rat plasma. In brief, aliquots (0.1 mL) of plasma were added to 10 µL of 0.1 N HCl. Then, plasma was added into 50 µL of IS (5 µg/mL in MeOH). After adding 500 µL of ACN, they were shaken for 10 min and centrifuged at 10,000× *g* for 5 min. Five hundred µL of the organic layer was transferred into a tube and dried at 40 °C under nitrogen stream. The residue was reconstituted in 50 µL of mobile phase, vortexed, and an aliquot was injected onto the HPLC system.

The HPLC system (Shimadzu, Kyoto, Japan) with ultraviolet detector was used for the determination of Met in plasma. Chromatographic separation assay was performed using a Luna SCX analytical column (150 × 4.6-mm inner diameter, 5-μm particle size, Phenomenex, Torrance, CA, USA) maintained at 30 °C. The mobile phase consisted of ACN:ammonium phosphate monobasic (10 mM) solution (10:90, *v*/*v*%) adjusted to pH 4 with orthophosphoric acid. Flow rate was 2 mL/min. For the UV detection of Met, the wavelength was set at 218 nm.

For the Met uptake study in HEK-293 cells overexpressing hOCT2, LC-MS/MS system was used. The HPLC system (Agilent 1290 system, Agilent Technologies, Santa Clara, CA, USA) was used with an Agilent 6495 triple-quad mass spectrometer controlled by MassHunter software. Mass spectrometer was maintained with a gas temperature of 200 °C, gas flow of 14 L/min, nebulizer pressure of 20 psi, capillary voltage of 3000 V, and cell accelerator voltage of 5 V. Positive multiple reaction-monitoring (MRM) was used to analyze Met using phenformin hydrochloride as an IS. For the separation of Met, a HILIC column (Kinetex HILIC, 4.6 × 50 mm, 2.6 μm) was used and maintained at 30 °C. Mobile phases consisted of 10 mM ammonium acetate in DW (pH 6.8):ACN (15:85, *v*/*v*%) and the isocratic mode was used with a flow rate of 0.5 mL/min.

### 2.11. Statistical Analysis

All data were expressed as mean ± standard error (S.E.) except the pharmacokinetic parameters. Statistical differences among groups were evaluated using a Student’s *t*-test and performed by Graphpad prism (Graphpad Software, La Jolla, CA, USA).

## 3. Results

### 3.1. Anti-Hyperglycemic Effect of Repeated Administrations of MLE

**Figure 1:**
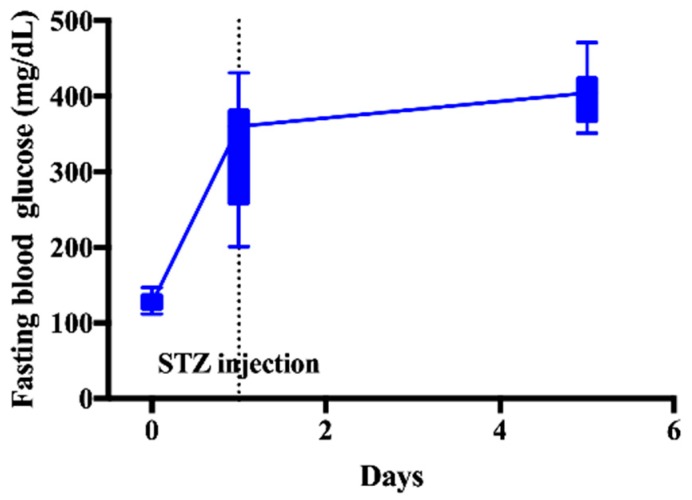
Fasting blood glucose levels after injection of streptozotocin (STZ) at a dose of 45 mg/kg in normal rats.

DM induced rats were used in all period of experiments. After the injection of STZ, hyperglycemia was induced and homogeneously maintained (Figure 1). Animals with the blood glucose level above 200 mg/dL were used in this study.

**Figure 2:**
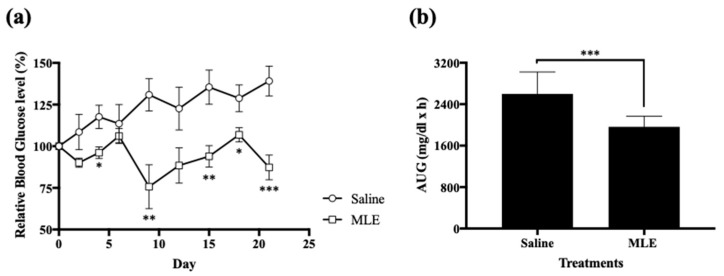
Relative blood glucose levels after oral administrations of Met (50 mg/kg) on day 22 in saline (circle) and MLE (square) treatments for 3 weeks (**a**), and AUG values of saline or MLE treatments for 3 weeks (**b**). * *p* < 0.05. ** *p* < 0.01. *** *p* < 0.001.

At 4, 9, 15, 18, and 21 day after administration of MLE, the relative blood glucose levels were significantly lowered as compared to those in saline treatment group (around 18, 42, 30, 17, and 37% at 4, 9, 15, 18, and 21 days, respectively) (Figure 2). During all periods of the experiment, relative blood glucose levels in MLE group were lower than those in the saline group (Figure 2a). Calculated AUGs were illustrated in Figure 2b. After the repeated administration of MLE for 21 days, AUGs in MLE group were significantly decreased by 24% compared with those in the saline group (Figure 2b).

### 3.2. Impact of Repeated Administrations of MLE on the Anti-Hyperglycemic Effect of Various Doses of Met

**Figure 3:**
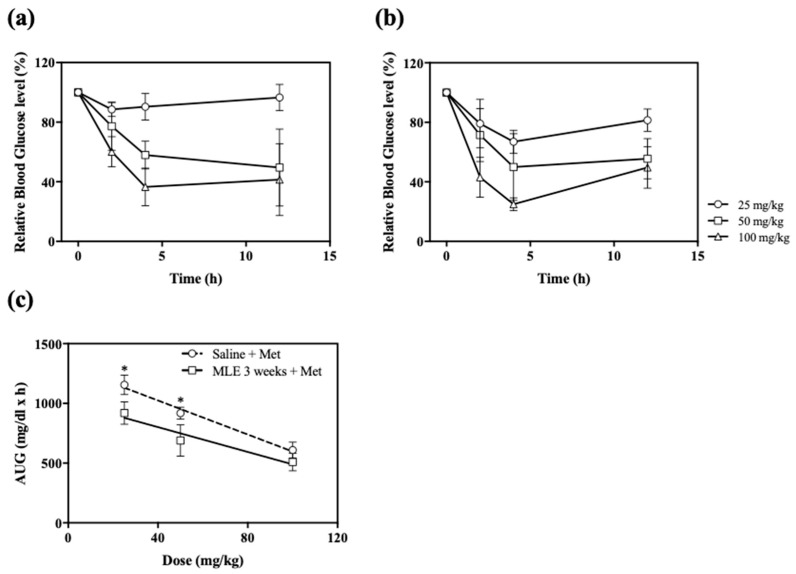
Relative blood glucose levels after oral administrations of Met (25, 50, and 100 mg/kg) on day 22 in saline (**a**) or MLE (**b**) treatments for 3 weeks, and AUG values of Met in saline (circle) and MLE (square) treatments for 3 weeks (**c**). * *p* < 0.05.

Time course of the relative blood glucose level after the oral administration of Met in the range 25–100 mg/kg in the saline or MLE treatment groups are illustrated in Figure 3. Anti-hyperglycemic effects of Met in the range 25–100 mg/kg showed a dose-dependent manner in either saline or MLE treatment groups (Figure 3a,b). In all experiment groups, the relative glucose level was decreased up to 4 h and maintained up to 12 h post-administration of Met. The mean relative glucose level values decreased to a maximum of 87.15 ± 4.25%, 60.76 ± 33.58%, and 36.59 ± 12.67% for 25, 50, and 100 mg/kg group, respectively, in the saline treatment group. In addition, these values decreased to a maximum of 66.99 ± 7.77%, 49.97 ± 22.44%, and 24.95 ± 4.33% for 25, 50, and 100 mg/kg group, respectively, in the MLE treatment group. AUG values after the administrations of different doses of Met (25, 50, and 100 mg/kg) are represented in Figure 3c. In two treatment groups (25 and 50 mg/kg), AUG values of MLE groups were significantly higher than those of saline groups. When the MLE was treated for 3 weeks, the AUG values were decreased by 20, 25, and 16% for 25, 50, and 100 mg/kg treatment groups, respectively, if compared to the saline treatment group (Figure 3c). The slope of the MLE treatment group was increased to −5.20 if compared to that of the saline treatment group. It suggests that the repeated administrations of MLE potentiated the anti-hyperglycemic effect of Met.

### 3.3. Influence of Repeated Administrations of MLE to the Pharmacokinetics of Met

**Figure 4:**
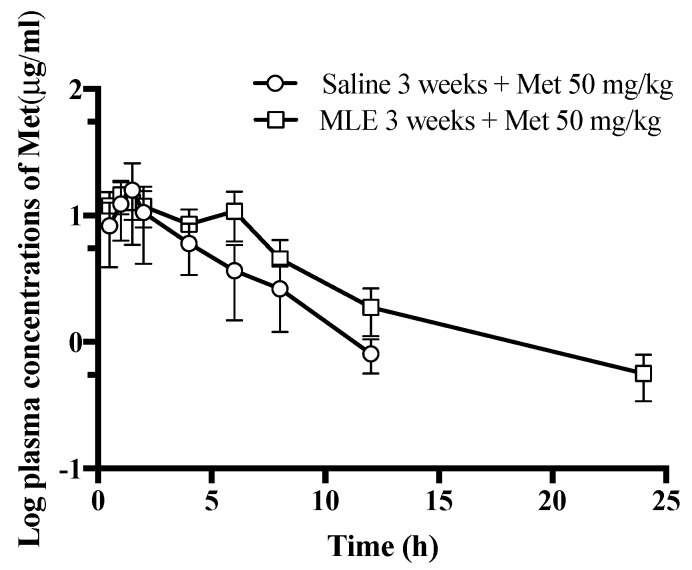
Plasma concentrations of Met after oral administrations of Met at a dose of 50 mg/kg in saline (circle) and MLE treatment groups (square).

To evaluate the influence of repeated administrations of MLE to the pharmacokinetics of Met, the pharmacokinetic profiles of Met were assessed in rats orally received the MLE or saline. Figure 4 shows the log plasma concentration–time curve of Met after administration of Met (50 mg/kg, PO) in saline and MLE groups, and the relevant pharmacokinetic parameters are listed in Table 1. Met in saline and MLE treated rats were detectable up to 12 and 24 h, respectively. After administration of Met, the absorption of Met between groups showed a similar pattern with T_max_ mean values of 1.7 and 1.9 h, respectively. These results were supported by the previous study wherein reported the slow absorption (T_max_, range 1.5–2.0 h) of Met in DM rats [31]. The T_1/2_λz was quite similar between the saline and MLE treatment group in the range of about 3–4 h. This is in line with previous studies in which the T_1/2_λz was ranged from 3 h to 5 h [31,32]. Mean AUC_0–24_ in MLE group was 1.7-fold larger than that in the saline group. However, there was no significant difference between groups. This phenomenon might be relevant to individual variability. The Cl/F in MLE group was significantly decreased by 50% compared to that in the saline group. This result indicates that the extended MLE treatment was reduced the elimination rate of Met.

### 3.4. Interference of MLE to Met Analysis in Rat Plasma

**Figure 5:**
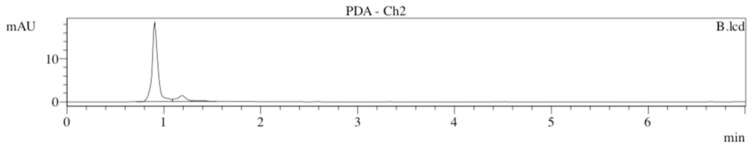
Chromatogram of blank plasma sample after the extended intake of MLE.

Before the pharmacokinetic analysis of Met, the influence of MLE to Met analysis in rat plasma was evaluated by adding the MLE to the Met spiking plasma. Accuracy (RE) and precision (RSD) values of inter- and intra-day were listed in Table 2. The intra-day RSD and RE were 9.29% and 7.65% for LQC, 8.47% and 8.48% for MQC, and 3.01% and 2.17% for HQC, respectively. The inter-day RSD and RE were 7.17% and 9.87% for LQC, 9.60% and 8.91% for MQC, and 5.03% and 3.77% for HQC, respectively. When the rat plasma with MLE (10 µg/mL) was used for the spiking of Met, the intra-day RE and RSD were 9.20% and 9.63% for LQC, 8.11% and 7.82% for MQC, and 2.49% and 2.03% for HQC, respectively. The inter-batch RSD and RE of all QC samples in MLE spiked plasma were ranged from 4.13% to 8.72%, and from 3.14% to 8.62%, respectively. These data were within the acceptance criterion recommended by US Food and Drug Administration [33]. In addition, after the exposure of MLE for 21 days, there is no detectable peak at the retention time of Met (Figure 5). It suggests that the MLE in rat plasma did not affect the Met analysis.

### 3.5. Effects of MLE on Met Uptake in HEK-293 Cells Overexpressing hOCT2

**Figure 6:**
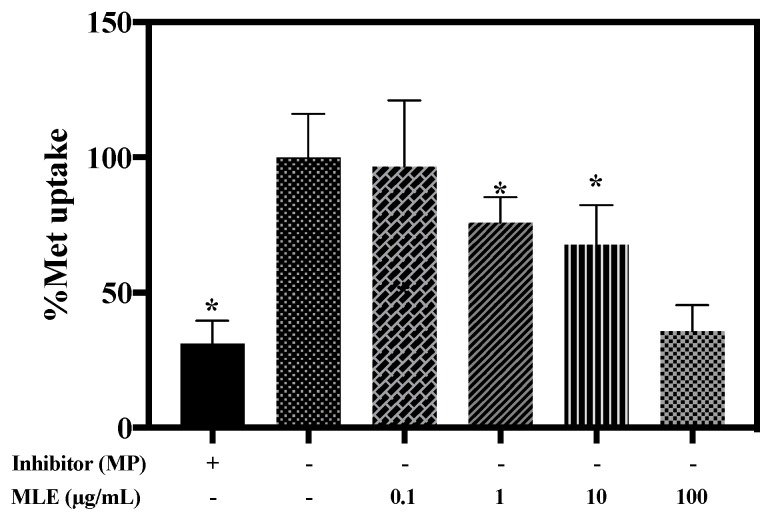
Met uptake with or without MLE in HEK-293 cells overexpressing hOCT2. * *p* < 0.05.

After the repeated administrations of MLE, the elimination rate of Met was lowered compared to the saline group. It has been reported that Met was predominantly transported to the kidney via hOCT2 for the elimination [34]. Thus, we postulated that the lowered elimination of Met is caused by the hOCT2 inhibition via the extended intake of MLE. Herein, the effect of MLE on Met uptake was evaluated in HEK-293 cell overexpressing hOCT2. Figure 6 was illustrated the change in Met uptake via hOCT2 by the various concentrations of MLE. MP, a well-known hOCT2 inhibitor, was significantly reduced Met uptake if compared to that of control. Dose-dependent inhibition of Met uptake was shown by increasing the concentration of MLE. Treatments of MLE at 1, 10 and 100 µg/mL were significantly decreased Met uptake to 36%, 68%, and 76%, respectively. In the MLE treatment group at 0.1 µg/mL, the Met uptake was similar to that of the control group. This result indicates that the MLE reduced the Met uptake via hOCT2 inhibition with the MLE concentration-dependent manner.

### 3.6. Hepatotoxicity of the Extended Intake of MLE

Serum biochemical parameters including alkaline phosphatase (ALP), alanine aminotransferase (ALT) were evaluated and listed in Table 3. There is no significant difference between ALP and ALT for control and MLE treated group. According to Han et al. (2010) [35], ALP and ALT values in normal SD rats were ranged from 91 to 250 U/L and from 26 to 101 U/L, respectively. It suggests that the dosage used in this study did not produce a negative impact on the liver.

## 4. Discussion

Evaluation of drug interactions is essential to applications of drugs and herbal medicines in clinical situations. In general, herbal medicine or health supplements are taken long-term. Thus, the extended influence of MLE to Met was evaluated in terms of pharmacokinetics and pharmacodynamics. This is the first study to evaluate the extended influence of MLE to Met.

In the present study, the extended intake of MLE (3 weeks) lowered the blood glucose level in DM-induced rats. This result is similar with previous literatures reported that the blood glucose lowering effects of the mulberry, leaf, and fruit extract [1,6,7,8,9,10,11,13,36,37,38,39]. These effects could be due to the presence of trans-caffeic acid, syringaldehyde, and chlorogenic acid in mulberry [11,13,40]. In particular, chlorogenic acid suppresses hepatic gluconeogenesis via both the inhibition of glucose-6-phosphatase (G6Pase) activity and the activation of AMP-dependent kinase (AMPK) [11,12]. In addition, it is reported that the trans-caffeic acid has ability to increase glucose uptake in muscle and GLUT-4 expression in skeletal muscles [30]. MLE used in this study includes the trans-caffeic acid, syringaldehyde and chlorogenic acid, thus results in this study were also supported by the previous investigations reported the anti-hyperglycemic effect of trans-caffeic acid, rutin, and chlorogenic acid in DM rats [11,12].

The extended intake of MLE (3 weeks) was associated with a 24% increased anti-hyperglycemic effect if compared with the saline treatment group. When the MLE was administered for 3 weeks, the anti-hyperglycemic effect of Met was enhanced in all doses of Met. Met treatment with MLE intake led to an enhanced increase in the anti-hyperglycemic effect. This increase indicates that the extended intake of MLE enhanced the anti-hyperglycemic effect of Met. Enhanced anti-hyperglycemic effect of Met by MLE was shown in all doses of Met, except the high dose of Met (100 mg/kg). This phenomenon might be due to the saturation of Met by the overdose [41]. In humans, the therapeutic dose of Met is 850 mg, corresponding to a dose of ~85 mg/kg in rats. Thus, a high dose of Met (100 mg/kg) in this study could result in the maximum anti-hyperglycemic effect of Met.

This enhanced effect could not be explained by only the individual anti-hyperglycemic effect of MLE. Interactions between herbal extract and anti-hyperglycemic drug have been reported to affect either efficacy or pharmacokinetics of the drug [20,21,22]. For instance, *Aloe vera* and *Andrographis paniculata* showed the inhibitory effects on CYP3A4 resulting in the increased efficacy of anti-hyperglycemic drugs such as pioglitazone, repaglinide, and glimepiride [28,42]. In opposition to this, St John’s Wort and Ginseng stimulated the induction of CYP3A4 resulting in the decreased efficacy of anti-hyperglycemic drugs, such as glibenclamide and pioglitazone [21,27]. Thus, we speculated that the extended MLE intake might alter the pharmacokinetic property of Met.

The bioanalytical method for Met was partially validated in the presence or absence of MLE in rat plasma. Whether the MLE was added or not in rat plasma, the inter- and intra-batch precision and accuracy of Met were estimated below 15%, which is the acceptance criteria recommended by US Food and Drug Administration [33]. It suggests that the presence of MLE in rat plasma did not impact on the bioanalytical method of Met.

Herein, the extended intake of MLE lowered the Cl/F of Met. This extended Met elimination phase suggests that the extended MLE intake altered the pharmacokinetics of Met in DM-induced rats. This pharmacokinetic feature could be explained by a hypothesis. It is demonstrated that Met relies on facilitated transport by transporters for delivery to the organs or tissues [43]. In particular, OCTs, including OCT1 and OCT2, and multidrug and toxin extrusion proteins (MATEs), which function as transporters of metabolic and xenobiotic organic cations, are known as determinants of Met response [43]. Met is delivered to the kidney by OCT2 and excreted in urine by MATEs. In this study, MLE was inhibited the hOCT2 in a concentration-dependent manner. When the MLE at 100 µg/mL was treated to the HEK-293 cells overexpressing the hOCT2, hOCT2 inhibition of MLE was similar to that of MPP, a hOCT2 inhibitor. These results suggest that the MLE intake could lower the elimination rate of Met by reducing the uptake of Met to the kidney. However, the levels/expression of the hOCT2 and MATEs in vivo system should be evaluated to clarify the result. Several cases of interactions between Met and herbal medicines have been reported that focus on the impact on transporters [23,44]. According to You et al. (2018), *Houttuynia cordata* ethanol extract reduced the rat OCT2-mediated renal excretion of Met and increased systemic exposure of Met. Also, there is another hypothesis. Met is metabolized by hepatic CYP2C11, 2D1, and 3A1/2, and eliminated via the kidneys in rats [30]. In studies, plant extracts of caffeic acid and chlorogenic acid could inhibit the activities of CYP2C11 and CYP3A1 [45,46]. Slowed elimination pattern of Met through the extended MLE intake might be explained by the decreasing hepatic metabolism of Met via inhibition of hepatic enzymes. However, further interaction studies between MLE and transporters are needed to clarify this issue.

## 5. Conclusions

This study is the first published report of the pharmacokinetic alternation of Met through 3 week-intake of MLE. MLE intake for 3 weeks produced an anti-hyperglycemic effect. Orally administered Met after the MLE intake for 3 weeks enhanced the anti-hyperglycemic effect of Met compared to Met alone. This enhanced effect of Met might be due to the increase of systemic exposure of Met. In addition, extended MLE intake altered the pharmacokinetic properties of Met via the reduction of Met uptake to kidney by inhibiting the hOCT2. Therefore, the extended MLE intake with Met could reduce the unwanted side effects of Met by decreasing the dose of Met.

## Figures and Tables

**Table 1:** Pharmacokinetic parameters after oral administrations of Met at a dose of 50 mg/kg in MLE or saline treatment groups.

Parameters	Unit	Saline 3 Weeks + Met	MLE 3 Weeks + Met
T_1/2_λz	h	3.36 ± 1.11	4.4 ± 1.27
T_max_	h	1.7 ± 1.3	1.9 ± 2.3
C_max_	μg/mL	10.85 ± 9.66	12.17 ± 1.06
AUC_0–24_	h μg/mL	42.74 ± 33.29	73.75 ± 15.23
V/F	mL/kg	6324.98 ± 2915.05	4143.72 ± 1291.5
Cl/F	mL/h/kg	1381.29 ± 545.57	657.74 ± 150.25 **

T_1/2_λz, half-life of the terminal portion of the curve; T_max_, time at the maximum drug concentration; C_max_, maximum plasma drug concentration; AUC_0–24_, area under the curve from 0 to the last; V/F, volume of distribution during the elimination phase; Cl/F, body clearance during the elimination phase. ** *p* < 0.01.

**Table 2:** Intra- and inter-day accuracy and precision of Met in rat plasma spiked with MLE.

Condition	QC	Intra-Day (*n* = 6)		Inter-Day (*n* = 5)	
		RSD%	RE%	RSD%	RE%
Met in rat plasma	LQC	9.29	7.65	7.17	9.87
	MQC	8.47	8.48	9.6	8.91
	HQC	3.01	2.17	5.03	3.77
Met in rat plasma spiked with MLE	LQC	9.2	9.63	6.35	6.01
	MQC	8.11	7.82	8.72	8.62
	HQC	2.49	2.03	4.13	3.14

**Table 3:** Serum biochemistry parameters in female and male rats treated with MLE.

Parameters	Units	Groups	
		Control Group	MLE Group
ALP	U/L	142.5 ± 10.2	145.3 ± 20.8
ALT	U/L	40.4 ± 10.3	42.0 ± 9.5

ALP, alkaline phosphatase; ALT, alanine aminotransferase.
